# Laser-induced rotation and cooling of a trapped microgyroscope in vacuum

**DOI:** 10.1038/ncomms3374

**Published:** 2013-08-28

**Authors:** Yoshihiko Arita, Michael Mazilu, Kishan Dholakia

**Affiliations:** 1 SUPA, School of Physics & Astronomy, University of St Andrews, North Haugh, St Andrews, KY16 9SS, UK

## Abstract

Quantum state preparation of mesoscopic objects is a powerful playground for the elucidation of many physical principles. The field of cavity optomechanics aims to create these states through laser cooling and by minimizing state decoherence. Here we demonstrate simultaneous optical trapping and rotation of a birefringent microparticle in vacuum using a circularly polarized trapping laser beam—a microgyroscope. We show stable rotation rates up to 5 MHz. Coupling between the rotational and translational degrees of freedom of the trapped microgyroscope leads to the observation of positional stabilization in effect cooling the particle to 40 K. We attribute this cooling to the interaction between the gyroscopic directional stabilization and the optical trapping field.

## Abstract (web summary)

Quantum state preparation of mesoscopic objects is a powerful tool for the study of physics at the limits. Here, Arita *et al*. realise the optical trapping of a microgyroscope rotating at MHz rates in vacuum where the coupling between the rotational and translational motion cools the particle to 40 K.

## Graphical abstract

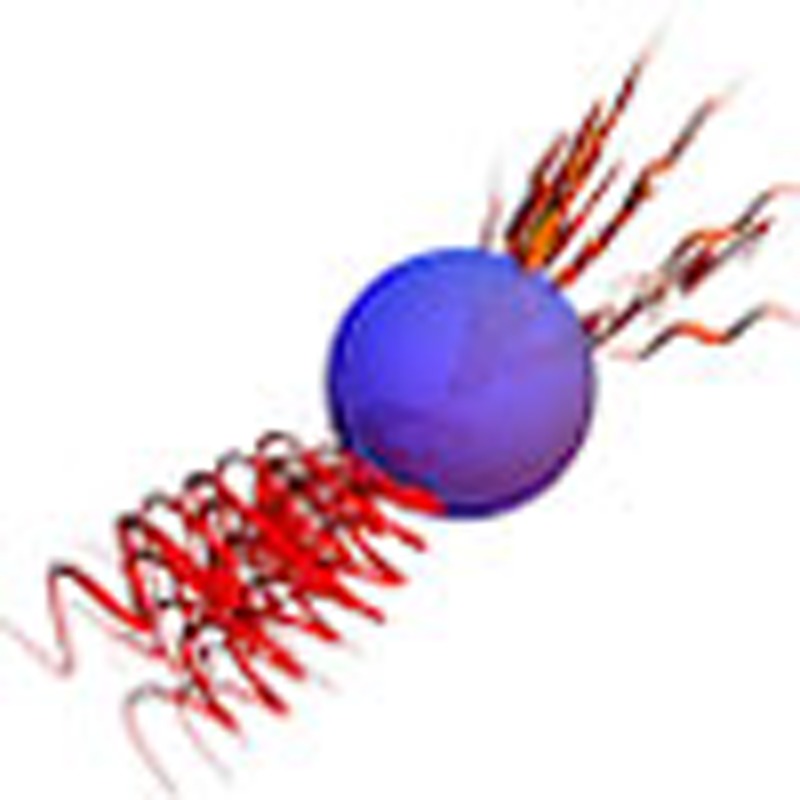

Exquisite control over all the degrees of freedom of a macroscopic object is a precursor for exploring the transitions between the classical and quantum world. With this remit, the complete optical control over an isolated nano- or microscopic particle in vacuum is an exciting testbed for such studies, crucially minimizing decoherence^1^. Such a system promises precision measurements of weak forces at the quantum limit solely constrained by the homogeneous linewidth of the ground state of the mechanical oscillator^2^,^3^,^4^,^5^,^6^. This raises the prospect of quantum entanglement between the incident light field and the mechanical modes of a trapped oscillator^6^,^7^,^8^,^9^,^10^, for example. Furthermore, there is the potential for exploring the predictions of the Casimir force and quantum friction^11^,^12^ if such a particle can be set into rotation at sufficiently high angular velocities and controllably positioned next to a surface.

Here, we present a trapped, levitated rotating microparticle confined in an optical potential, rendering it well isolated from the thermal environment. Previous studies have shown that microparticles may be held in vacuum and then cooled to millikelvin temperatures using ‘active’ feedback schemes^13^,^14^. However, by including the rotational degrees of freedom^15^,^16^, we can consider cooling in the absence of any ‘active’ feedback, in contrast to previous experiments^13^,^14^. This approach opens up a powerful route to explore the fascinating predictions of quantum friction^11^,^12^, which may ultimately have a profound impact upon nanoscale devices. We demonstrate controlled rotation of a single trapped microparticle at background gas pressures ranging from 10^5 ^Pa (atmospheric) down to 10^−1 ^Pa, where we recorded the maximum rotation rates of up to *f*_rot_=5 MHz (increasing to 10 MHz for short durations of time). We observe coupling between the rotational and translational degrees of freedom of the trapped object that leads to particle effective-cooling to 40 K in the absence of any ‘active’ cooling method. This rotating particle may be considered as a microscopic gyroscope.

## Results

### AM transfer

When a birefringent uniaxial crystal, such as vaterite (see Methods for details), is trapped in a linearly polarized (LP) beam, the optical axis of the crystal will align with the electric field, which is perpendicular to the propagation direction of the beam. In contrast to LP light, circularly polarized (CP) light incident on a birefringent particle will induce a rotation of the particle^17^,^18^. This is caused by the transfer of spin AM from the beam to the microparticle. Indeed, the birefringent particle induces a phase retardation between the ordinary and extraordinary components of the beam reversing the photon AM (±*ħ* per photon) of the incident CP light. If the particle acts as a half-wave (λ/2) plate, the maximum spin AM will be transferred to the particle, with a spin AM change of 2 *ħ* per photon. Considering the conservation of energy and AM in this process, a frequency shift arises owing to the angular Doppler effect as (*ω*_1_−*ω*_2_)/2=Ω_rot_=2*πf*_rot_, where *ω*_1_ and *ω*_2_ are the angular frequencies of the light before and after passing through the particle, repectively, and Ω_rot_ is the angular rotation frequency of the particle^19^. As a result, we observe optical beating at 2Ω_rot_ when the particle rotates at Ω_rot_.

### Power spectra

**Figure 1:**
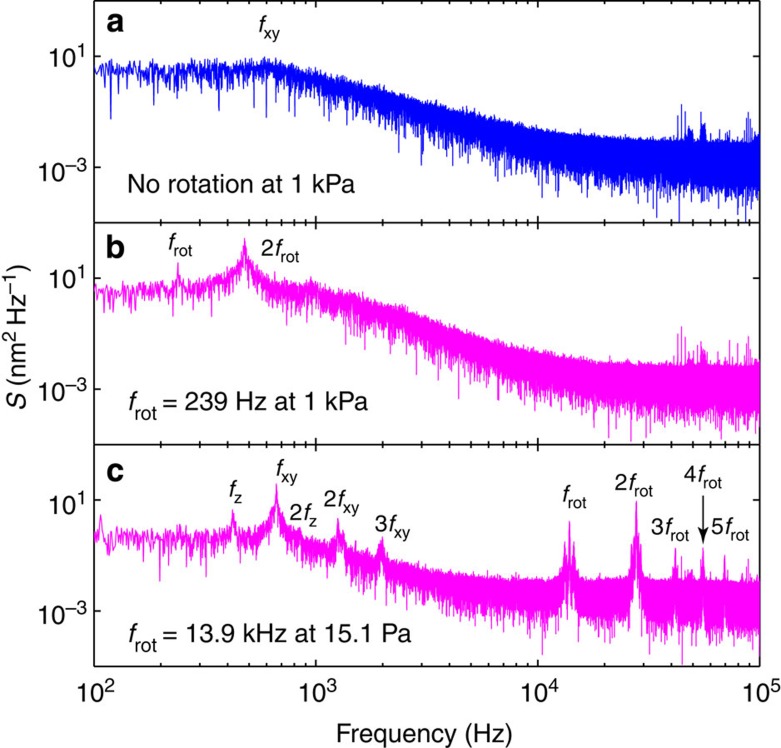
PSD signals of the scattered light from a trapped particle. (**a**) Optical trapping by an LP light field (that is, no induced rotation) at 1 kPa. (**b**) Optical trapping by CP light at 1 kPa showing an optical beating frequency at 2*f*_rot_ together with *f*_rot_. (**c**) PSD at 15.1 Pa showing translational (*f*_xy_, *f*_z_) and rotational (*f*_rot_) frequencies of a trapped particle.

Figure 1 shows typical power spectral density (PSD) signals (measured by PD_i=1_ as defined in the Methods section) of the scattered light from a trapped particle. In Fig. 1a, the particle experiences a rotational trapping torque owing to an LP light field. Figure 1b, c show the cases when the particle is trapped by a CP light field at pressures of 1 kPa and 15.1 Pa, respectively. In the following, we consider the PSD defined using the Fourier transform of the signal measured by PD_i_ and denoted as *S*(*f*). The PSD measured at low pressures exhibits resonance peaks at the translational oscillation frequencies of *f*_xy_ and *f*_z_ corresponding to the periodic motion of the particle in the lateral *x*–*y* and axial *z* directions. Figure 1c shows the oscillation frequencies of *f*_xy_ at 660 Hz and *f*_z_ at 420 Hz for this specific particle. Further, the PSD shows an optical beating at 2*f*_rot_ when the particle rotates at a rate of *f*_rot_ (Fig. 1b, c). In practice, *f*_rot_ is also detected because of the variation in the photodiode signal induced by small optical asymmetry of the particle. We remark that higher harmonics of the fundamental rotation frequency of *f*_rot_ and translational oscillation frequencies of *f*_xy_ and *f*_z_ are also observed in the PSD signal (Fig. 1c).

### Rotation rate

**Figure 2:**
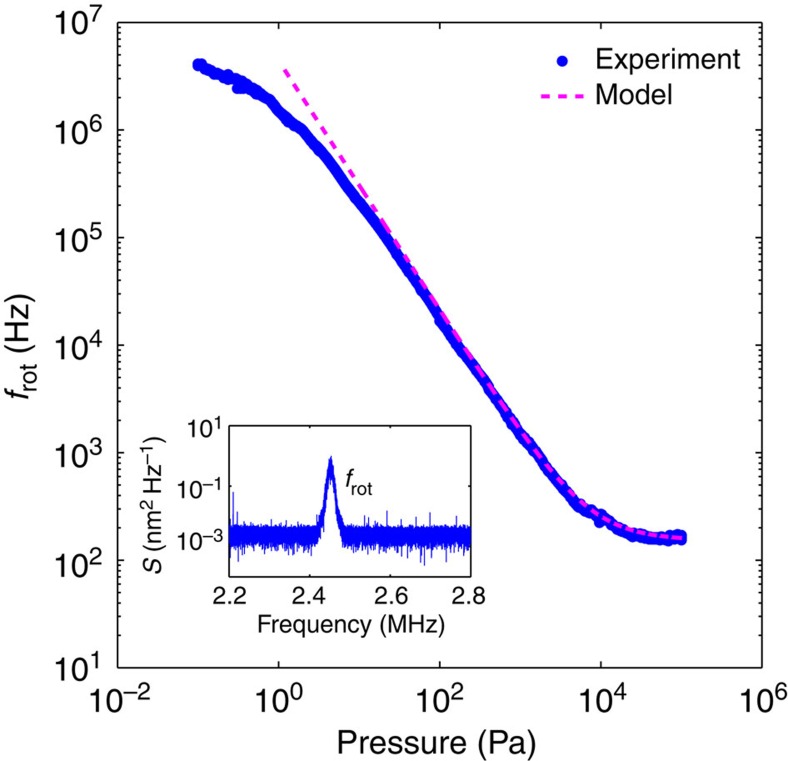
Rotation rate of a trapped particle at different gas pressures. The model fits to the experimental data for 0≤*K*_n_≤880. Inset shows the PSD at a rotation rate of 2.45 MHz at a pressure of 1 Pa.

A trapped spinning birefringent microparticle will reach a terminal rotation frequency owing to the Stokes drag torque, dependent upon particle size and gas viscosity. The effective gas viscosity, *μ*_*e*_ experienced by a spherical microparticle can be empirically estimated as^20^


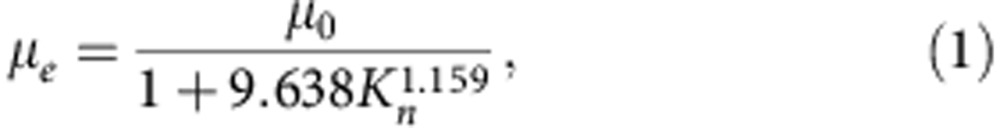


where *μ*_0_ is the viscosity coefficient at the reference pressure *P*_0_, and *K*_*n*_=λ_*a*_/*d* is the Knudsen number. Here 
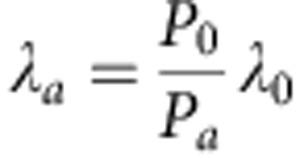
 is the mean free path (mfp) of air molecules at a pressure *P*_*a*_ relative to the mfp of *λ*_0_ at the reference pressure *P*_0_, and *d* is the diameter of the particle. As the pressure decreases, the mfp of the surrounding gas molecules becomes comparable to the particle diameter implying *K*_*n*_≈1. This marks a transition to a regime where radiometric forces are negligible and the viscosity becomes proportional to the pressure. Figure 2 shows the rotation rate *f*_rot_ of a trapped particle as a function of pressure measured by observation of the PSD (Fig. 2 inset). An initial rotation rate of 110 Hz is recorded at atmospheric pressure, which increases to a stable rotation rate of 5 MHz for a pressure of 0.1 Pa. Decreasing the pressure further can lead to rotation rates of up to 10 MHz, although, at such rates, the particle is lost in a short period of time. We remark that this represents, to date, the largest measured rotation rate for a ‘man-made’ object^21^. The model in Fig. 2 is calculated using equation (1), implying a pressure-dependent Stokes rotational drag coefficient. The discrepancy between the model and experimental values at pressures below 10 Pa could be attributed to particle instability induced by heat because of light absorption^22^,^23^, while the low pressure particle loss might be due to the large inertial forces experienced by the particle at high rotation rates.

### Parametric coupling

**Figure 3:**
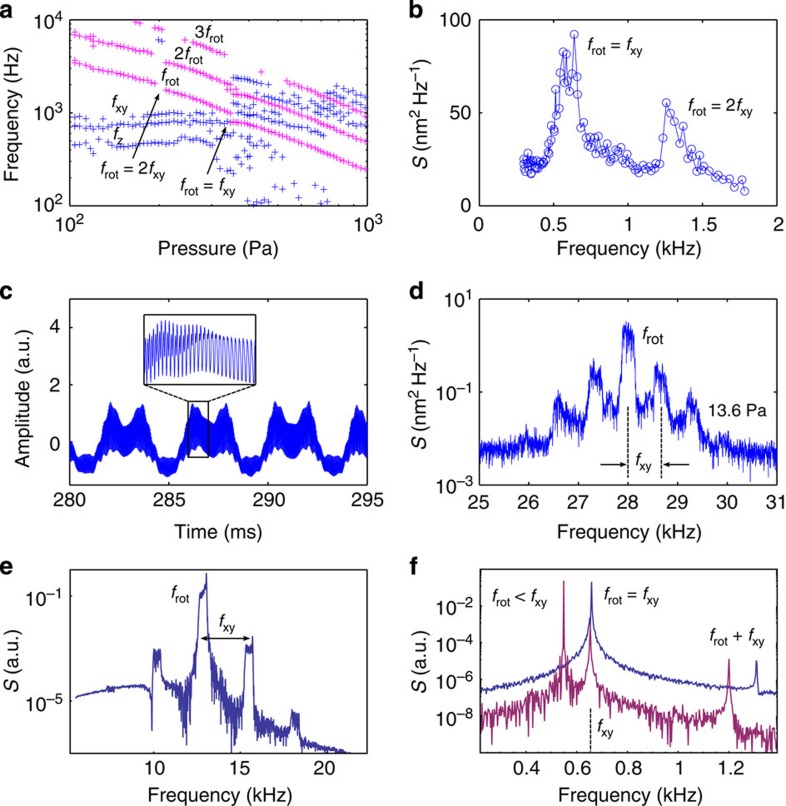
Coupling of the rotational and translational motion of a trapped particle. (**a**) Major peaks of the PSD signal around the resonance frequency at *f*_rot_≈*f*_xy_. In red are frequency peaks associated with rotation and in blue the ones associated with translational oscillations. (**b**) Resonances found at *f*_xy_ and 2*f*_xy_ when *f*_rot_ scans across these frequencies. (**c**) Photodiode signal in time domain showing mixed frequency components at 13.6 Pa. Inset shows the expanded view of the selected region. (**d**) Fourier transform of the time-domain signal (Fig. 3c) showing the rotation frequency of *f*_rot_ with sidebands separated by *f*_xy_. (**e**) Simulated PSD signal at a high rotation frequency showing the appearance of sidebands and their harmonics due to the modulation of the trapping frequency. (**f**) Simulated PSD signal for two different gas viscosities corresponding to the resonant (blue) and non-resonant (red) cases.

An optically trapped and simultaneously rotated particle in vacuum offers original perspectives on particle dynamics. It is of particular interest to study the dynamics when the rotation frequency *f*_rot_ coincides with the oscillation frequency *f*_xy_ of the trapped particle. A series of power spectra tracking the major peaks of the PSD at each pressure from 1 kPa to 100 Pa are shown in Fig. 3a. The rotation frequency signal exhibits kinks at *f*_rot_≈*f*_xy_ (at 380 Pa) and at *f*_rot_≈2*f*_xy_ (at 210 Pa). Figure 3b shows the amplitude of the PSD peak at the oscillation frequency *f*_xy_ as the rotation frequency of the trapped particle changes. We observe an enhanced signal at *f*_rot_≈*f*_xy_ corresponding to a driven resonance. A second resonance occurs when *f*_rot_≈2*f*_xy_, suggesting a parametric resonance^24^. The coupling between the oscillatory motion and rotational motion of the particle can also be observed in Fig. 3c,d. The photodiode signal (PD_1_) in time domain measured at a pressure of 13.6 Pa exhibits fine rotational modulation (Fig. 3c inset), which is further modulated by slower frequency components (Fig. 3c). The power spectrum of these modulations reveals the rotation frequency *f*_rot_ accompanied with sidebands separated by *f*_xy_ (Fig. 3d).

We approximatively model the dynamics of a birefringent particle in an optical potential and subject to position- and orientation-dependent torque. To simplify the system while maintaining its main optical properties, we consider the induced polarization of an anisotropic dipole^25^ corrected for the anisotropic radiative process^26^. The optical forces and torques are calculated by generalizing the cycle-averaged Lorentz force^27^ and torque^28^ to account for the anisotropy owing to birefringence. Optically, the spherical aberration introduced by the total internal reflection at the glass–vacuum interface is taken into account by using angular spectral decomposition of the incident beam^29^ (Supplementary Note 1). We remark that at the levitated equilibrium position of the particle, the trapping forces can be approximated with an optical harmonic potential originating from a CP Gaussian beam. In the simulations, we use this potential and adjust the beam parameters such that the transversal trap oscillation is ≈660 Hz for a vaterite microparticle whose diameter is 4.40 μm. The rotation of the microparticle is modelled by the Euler equations for a solid sphere with a slight asymmetry of 0.1% in one of its moments of inertia (Supplementary Note 2). This mechanical asymmetry introduces a principal momentum axis that does not overlap with the optical axis of the particle.

For these mechanically anisotropic particles, the Brownian stochastic torques are introduced in the rotational Langevin equation^30^, which are generalized to include an external torque^31^ and contributions from the Euler equations in the body frame of reference (Supplementary Note 3). Finally, the detection is simulated by Fourier transforming the dipole polarization intensity along a fixed direction. This Fourier transform corresponds to the PSD signal observed. Figure 3e shows the simulated PSD of a rotating dipole particle oscillating periodically in the beam. The central resonance corresponds to the polarization change due to rotation, whereas the multiple sidebands correspond to periodic variations of the electric field strength as the particle oscillates in the trap. These simulations indicate that the coupling behaviour observed in Fig. 3b occurs when the rotational frequency is resonant with the fundamental and the second harmonic of the translational frequency corresponding, respectively, to a driven oscillator resonance and a parametric resonance. Indeed, parametric resonance occurs as the trap stiffness varies slightly for different orientations of the particle, whereas this orientation changes as the particle rotates. Figure 3f shows the enhancement of the PSD signal at the transversal oscillation frequency *f*_xy_ peak as the rotational frequency becomes resonant.

### Cooling

**Figure 4:**
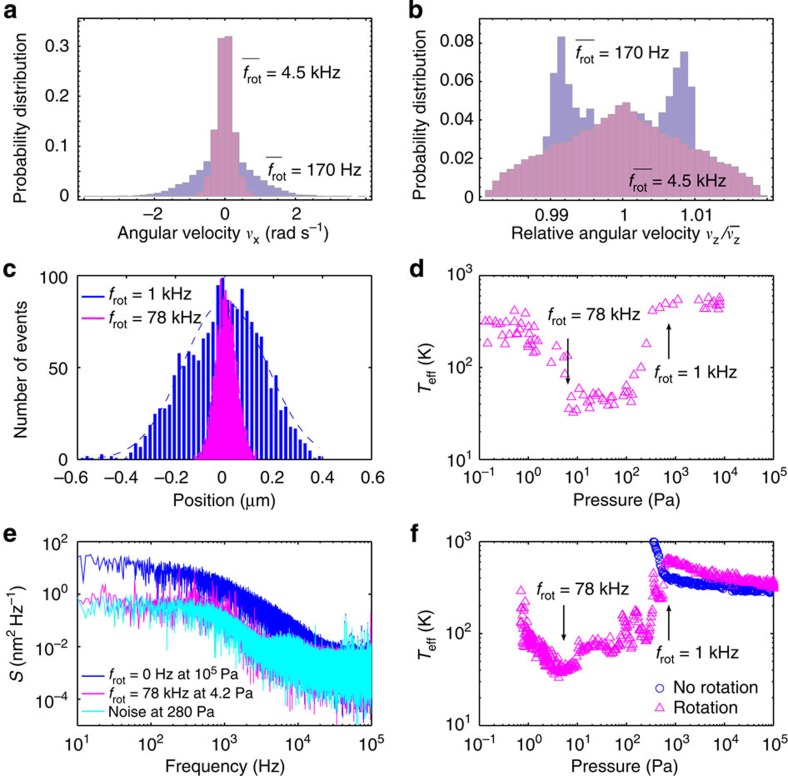
Effective cooling of the microgyroscope as a function of the rotation rate. (**a**) Simulated distribution of the angular velocity, ν_x_ around a transversal axis and (**b**) around the longitudinal axis, ν_z_ for different average rotation rates. The bimodal distribution is owing to particle precession at low rotation rates. (**c**) Position distributions of a trapped particle at different rotation rates. (**d**) Particle effective temperature, *T*_eff_ at different pressures determined by the equipartition method. (**e**) PSD signals of the scattered light from a trapped particle with and without rotation. (**f**) Effective temperatures, *T*_eff_ determined for both a rotating and a non-rotating particle using the PSD signals.

Our trapped microparticle has three rotational and three translational degrees of freedom. These are coupled due to the optical anisotropy of the microparticle, which can be seen in Fig. 3b,d–f. We now progress to investigate the impact of the rotation rate on the rotational degrees of freedom. In this case, we observe the effective cooling of microparticles through rotation in the absence of any ‘active’ feedback method. Akin to the motion of a spinning top, a rotating body offers inertial stiffness, which prevents the body from drifting from its desired orientation. Here the high rotation rates achieved using the microgyroscope lead to the intrinsic stabilization of its axis of rotations with respect to perturbations. This effect is similar to stabilizing the rigid-body dynamics of an oval football^32^ or spin-stabilized satellite^33^. Our numerical simulations indicate that for increasing rotation rate, the distribution width of the fixed-frame transversal angular velocities ν_x_ and ν_y_ decrease (Fig. 4a,b). This effect can be seen as a cooling effect on the two rotational degrees of freedom of the microgyroscope at the expense of the third rotational degree of freedom around which the rotation occurs, supporting our conclusions from the experimental data. Owing to rotational–translational coupling mentioned above, this rotational stabilization also enables cooling for all the three translational motion degrees of freedom. More specifically, when the laser is switched on, it heats the transversal motion of the particle due to the stochastic fluctuation of the microparticle orientation. As the particle rotates faster, its orientation is stabilized by the gyroscopic effect and as such the particle experiences relative cooling.

## Discussion

To investigate the cooling effect in more detail, we study the integral of the PSD signal *S*(*f*). This quantity is proportional to the mean square displacement of the particle 
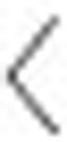
*x*^2^
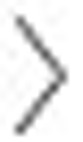
 and depends only upon trap strength and temperature^34^,^35^. That is


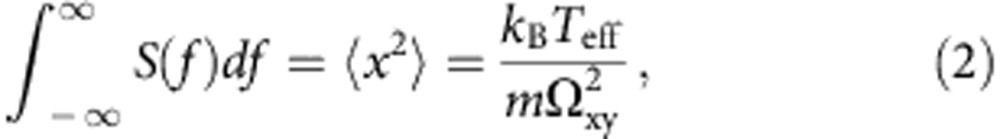


where *k*_B_ is the Boltzmann’s constant, *T*_eff_ defines the effective temperature, *m* is the mass of the particle, 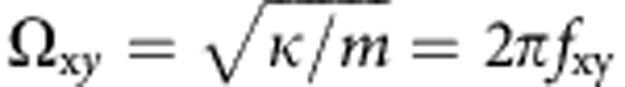 the natural oscillation frequency in absence of damping and *κ* denotes the trap stiffness (Supplementary Fig. S1 and Supplementary Note 3). A similar relationship can be obtained when considering the axial *z* direction. Equation (2) can be used in different ways to determine the effective temperature of the microparticle in the optical field. In this paper, we use three approaches. The first approach is based on the equipartition theorem illustrated in Fig. 4c that compares the distribution of particle positions in a lateral direction *x* for different rotation rates at *f*_rot_=1 kHz and at *f*_rot_=78 kHz. The *T*_eff_ of the particle is calibrated at room temperature by associating with the variance of the position distribution of the trapped particle held at atmospheric pressure. Figure 4d shows the *T*_eff_ of the trapped particle at different rotation rates, where we observe that the effective temperature of the particle reaches a minimum of 40 K at *f*_rot_=78 kHz. Similar cooling behaviour can be observed in the axial *z* direction (Supplementary Fig. S2).

The effective temperature can also be determined using the power spectral density of the scattered light field either through its integral (2) or by measuring its low frequency response (Supplementary Fig. S3 and Supplementary Note 3). Figure 4e compares the PSD signals for the cases of both a rotating and a non-rotating particle. The integral of this PSD signal is proportional to the centre-of-mass positional variance and is thus related directly to the particle effective temperature in a trapping potential. We monitor *T*_eff_ as a function of pressure for a rotating and a non-rotating particle (Fig. 4f). For the non-rotating particle, we do not observe any cooling effect. Additionally, the trap becomes unstable at 1 kPa and the particle is lost from the trap typically at 300 Pa, corresponding to the *f*_rot_≈*f*_xy_ resonance. Applying an optical torque stabilizes the particle and enables us to maintain the trapped particle through this resonant transition. The PSD signal shows that *T*_eff_ increases up to the resonance point (*f*_rot_≈*f*_xy_). At higher rotation frequencies, it is seen to decrease, reaching a minimum value of 40 K at a rotation frequency of *f*_rot_=78 kHz corresponding to a background pressure of 4.2 Pa. Altogether, all three methods determining the temperature lead to the observation of effective cooling of the centre-of-gravity motion of the particle. In future work, we will explore the origin of the particle instability at high rotation rates.

In conclusion, the trapped microgyroscope presents a powerful and original route to explore new directions in cavity optomechanics and is a major step towards measuring rotational quantum frictional forces in vacuum.

## Methods

### Experimental set-up

**Figure 5:**
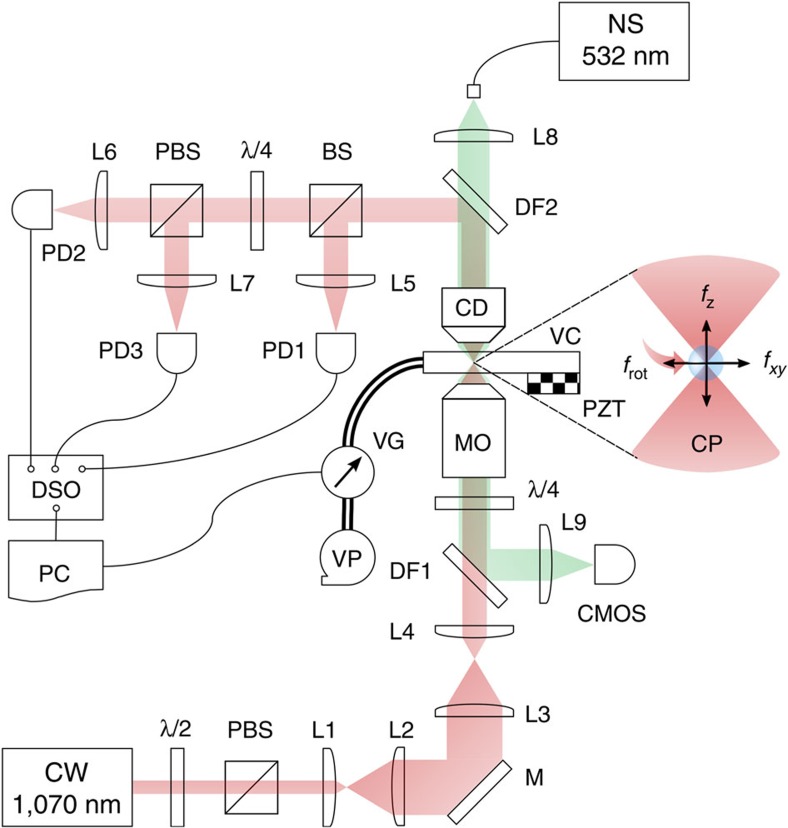
Schematic of the experimental set-up used for the trapping and rotation of a birefringent microparticle in vacuum. Labels denote the continuous wave (CW), half-wave plate (λ/2), polarizing beam splitter (PBS), lenses (L), mirror (M), dichroic filters (DF), quarter-wave plate (λ/4), microscope objective (MO), circularly polarized trapping laser beam (CP), condenser (CD), 50/50 beam splitter cube (BS), photodiodes (PD_i_), nanosecond laser (NS), fast imaging device (CMOS), digital storage oscilloscope (DSO), computer (PC), vacuum chamber (VC), piezo electric transducer (PZT), vacuum gauge (VG), vacuum pump (VP), rotational frequency of a trapped particle (*f*_rot_) and translational oscillation frequencies (*f*_xy_, *f*_z_) of a trapped particle in lateral and axial directions.

The experimental system (Fig. 5) comprises a miniature vacuum chamber (VC), which has two optical glass windows. Light is focused in the VC using a high numerical aperture microscope objective (MO, Nikon Ltd., E Plan × 100, NA=1.25). A piezo electric transducer (PZT) is affixed to the chamber to load the levitation air/vacuum trap with microparticles from the glass substrate^18^. Rotation of microparticles is achieved by trapping a birefringent spherical vaterite crystal (4.40 μm in diameter) with a CP light field at an optical power of 25 mW (measured at the back aperture of the MO). The polarization state of the scattered light from the trapped particle is recorded by fast photodiodes (PD_i_, i=1–3) to determine the particle dynamics.

### Beam preparation

The trapping laser beam (IPG Laser GmbH, YLM-5-1070-LP: continuous wave (CW), wavelength 1,070 nm, power 5 W) propagates through a half-wave plate (λ/2) followed by a polarizing beam splitter cube (PBS). This enables the control of the optical output power as well as the generation of a LP light field. The beam is collimated and expanded to overfill the back aperture of the MO in order to obtain a diffraction-limited focal spot. A quarter-wave plate (λ/4) is placed immediately before the MO to create a CP light field.

### Vacuum chamber

The VC is made of stainless steel with a volume of 27.7 μl. This has two optical glass windows (Harvard Apparatus Ltd., CS-8R: 8 mm in diameter, 150 μm in thickness) compatible with the microscope objective used. An annular piezo electric transducer (PZT, APC International Ltd., Cat. no.70-2221) affixed to the chamber is operated at 340 kHz to detach the microparticles from the lower glass window to load the optical trap in air/vacuum.

### Birefringent crystals

**Figure 6:**
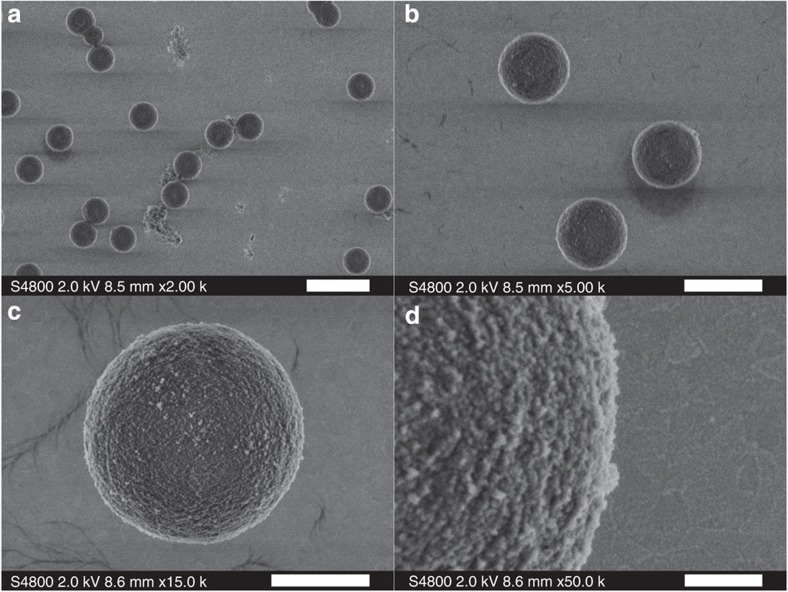
SEM images of vaterite crystals. (**a**) A selection of monodisperse particles. (**b**) A closer look at Fig. 6a. (**c**) A single vaterite crystal with a near-perfect sphericity. (**d**) A closer look at Fig. 6c showing the surface roughness of the particle. Scale bars, 10 μm in (**a**), 5 μm in (**b**), 2 μm in (**c**) and 500 nm in (**d**).

We synthesize vaterite crystals for our experiments^18^. Vaterite is a positive uniaxial birefringent material with a spherical morphology. Figure 6 shows the images of vaterite crystals acquired by a scanning electron microscope (SEM) at different scales. The obtained vaterite particles are monodisperse with a mean diameter of 4.40±0.05 μm (2σ) and a surface roughness of 27.6 nm (2σ).

### Detection scheme

Once a single vaterite sphere is trapped and rotated at atmospheric pressure, the chamber pressure is gradually reduced to 10^−1^ Pa. The transmitted light through the trapped particle is collected using a condenser lens (CD, Nikon Ltd., E plan × 10, NA=0.25 in air) and directed on fast photodiodes (PD_i_, Thorlabs Inc., DET10C, InGaAs) to record the particle dynamics. PD_2_ and PD_3_ measure the right and left CP components of the scattered light, which are used for the determination of the optical torque transfered to the particle^18^. A fast CMOS camera (Mikrotron GmbH, EoSens: >10 k fps) synchronized with nanosecond laser pulses (NS, Elforlight Ltd., SPOT: pulse width ≤1 ns) acts as a stroboscopic illumination to track the translational centre-of-mass motion of a trapped particle in *x*, *y* and *z* directions.

## Author contributions

All authors contributed to the development and planning of the project, interpretation and discussion of the data and the writing of the manuscript. Y.A. performed the experiments and data analysis, and M.M. the theory and numerical modelling. K.D. initiated and supervised the project.

## Additional information

**How to cite this article:** Arita, Y. *et al*. Laser-induced rotation and cooling of a trapped microgyroscope in vacuum. *Nat. Commun.* 4:2374 doi: 10.1038/ncomms3374 (2013).

## Supplementary Material

Supplementary Information Supplementary Figures S1-S3 and Supplementary Notes 1-3
