# Supplementary material for: Laser-induced rotation and cooling of a trapped microgyroscope in vacuum
Source: Nat Commun. 2013 Aug 28;4:2374. doi: 10.1038/ncomms3374 (PMC3763500; doi:10.1038/ncomms3374)
Supplement: Supplementary Information — Supplementary Figures S1-S3 and Supplementary Notes 1-3 [file ncomms3374-s1.pdf]

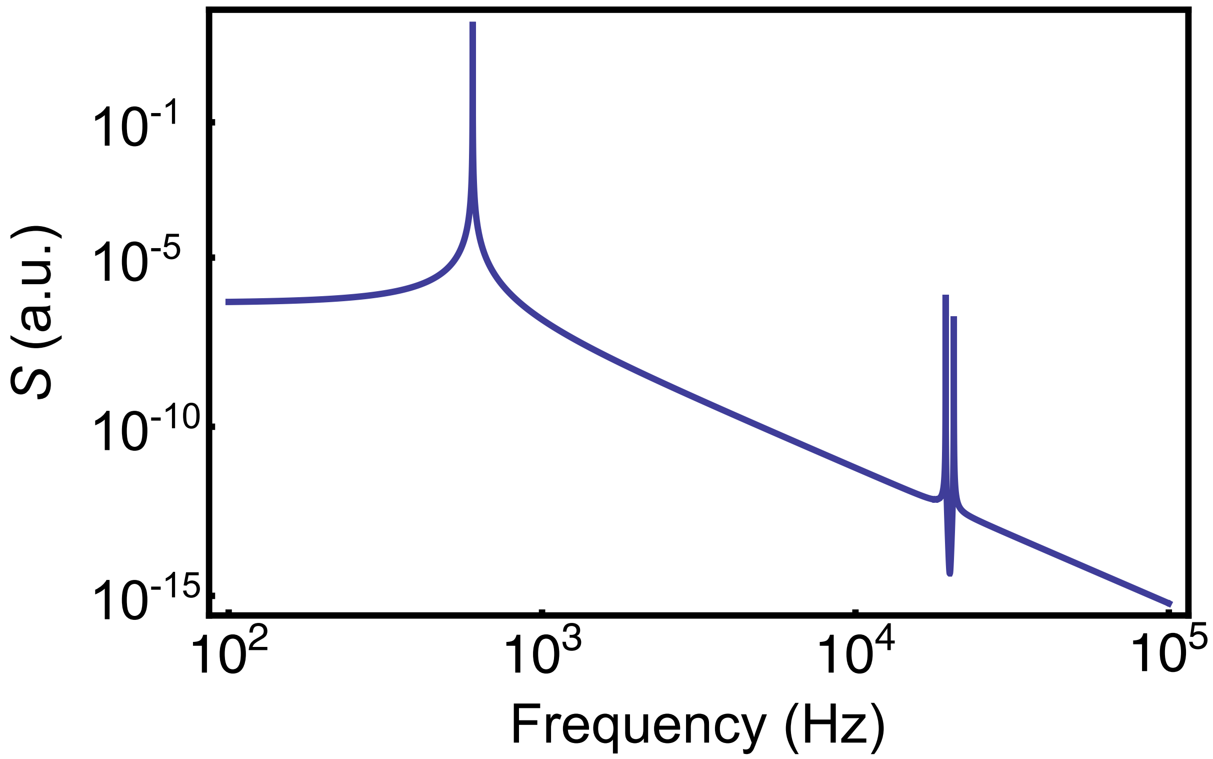

Supplementary Figure S1: **Numerical PSD simulation.** Example numerical simulation of the power spectral density, $S(f)$ from a trapped particle oscillating at $\Omega_0/(2\pi) = f_{\mathrm{xy}} = 600\mathrm{Hz}$ and subject to a periodic perturbation due to the rotation at $f_{\mathrm{rot}} = 20\mathrm{kHz}$.

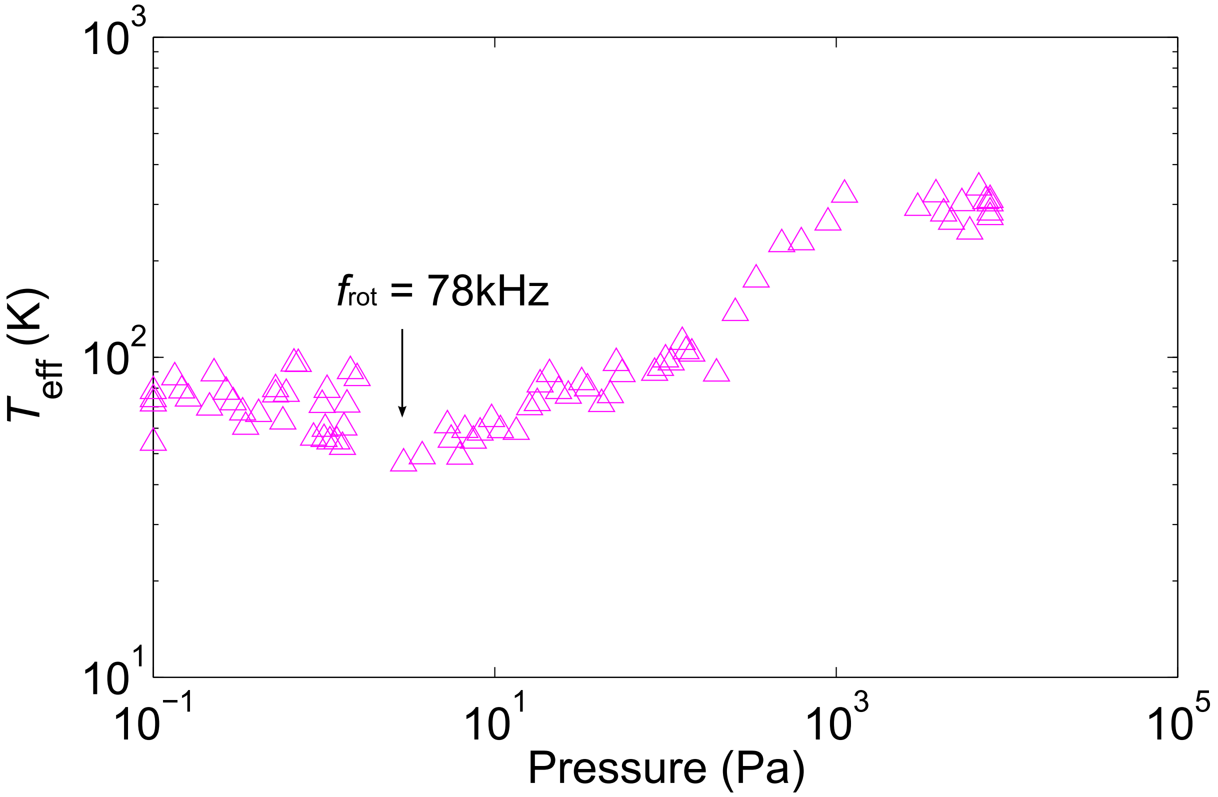

Supplementary Figure S2: **Axial particle effective temperature.** Effective temperature T_{eff} at different pressures determined by the equipartition method for the z-direction using the integrated intensity signals in the field of view of the fast CMOS camera. The variations of this intensity is linearly related to the particle z-displacement. We observe that the effective temperature reaches a minimum of 45K at f_{rot}=78kHz compatible with the measured minimal temperature of the transversal displacement (see Figure 4d in the main paper). This observation confirms that the rotation stabilizes and effectively cools the particle position in all three directions.

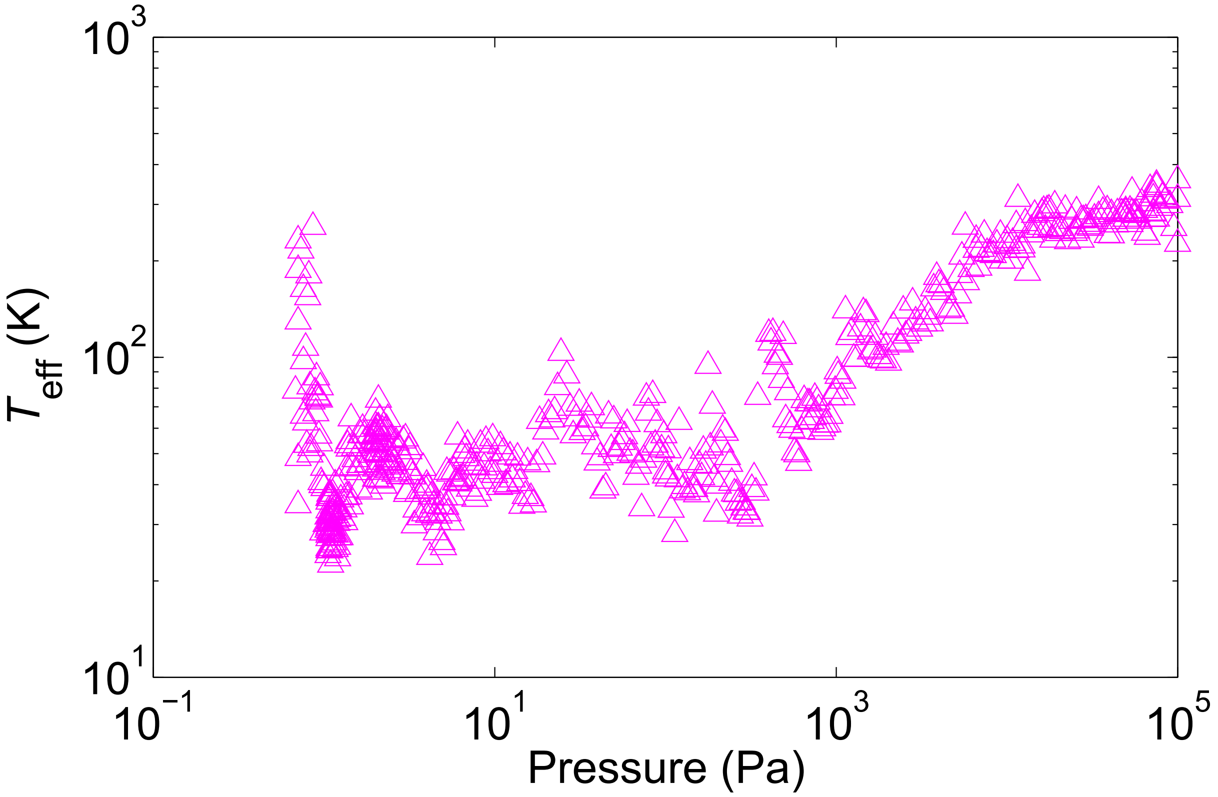

Supplementary Figure S3: **Low frequency limit temperature.** Effective temperature measured using the PSD signals at low frequency, $S(\Omega = 0)$, averaged between 1Hz and 50Hz at different pressures. As shown in equation (S19), temperature can also be determined by the PSD signal at low frequency, $S(\Omega = 0)$. This method delivers similar results to the integrated PSD approach (eq. (2) in the main paper).

Supplementary Note 1

Optical forces and torques on birefringent nanoparticles

For very small particles it is possible to model the overall scattering of a nanoparticle as the field originating from an anisotropic induced dipole. In the static case the polarisability tensor $\boldsymbol{\alpha}$ in vacuum is given by [25]:

$$\boldsymbol{\alpha}_0 = 3v(\boldsymbol{\varepsilon} - \mathbf{I})(\boldsymbol{\varepsilon} + 2\mathbf{I})^{-1} \tag{S1}$$

where $v = 4\pi a^3/3$ is the volume of a particle with a radius a, $\mathbf{I}$ the identity tensor and $\boldsymbol{\varepsilon}$ the permittivity tensor. The static polarisability needs to be corrected for the radiative process in order to fulfil the Optical Theorem [26] . The radiative correction leads in the case of spherical anisotropic particles to:

$$\boldsymbol{\alpha} = \boldsymbol{\alpha}_0 \left(\mathbf{I} + i\frac{k_0^3}{6\pi}\boldsymbol{\alpha}_0 \right)^{-1} \tag{S2}$$

where $k_0 = 2\pi/\lambda$ is the vacuum wave vector. The sign of the wavevector corresponds to a monochromatic wave proportional to $\exp(i\omega t)$. In the absence of birefringence, all tensors become scalar quantities and the resulting scalar polarisability including radiative corrections leads to the isotropic case.

For an induced anisotropic dipole defined by $P_j = \alpha_j^n E_n$, we can calculate the optical cycle-averaged Lorentz force by generalising the approach described in [27]

$$< F^i >= \frac{1}{2}\Re e \left(\alpha_j^n E_n \partial^i (E^j)^* \right) \tag{S3}$$

where E_n is the electric vector field and where summation over repeating indices has been assumed.

In the same way, we can generalise the torque acting on the dipole [28] for the anisotropic case as

$$< T^i >= \frac{1}{2}\Re e \left(\alpha_j^n E_n \epsilon^{ijk} (E_k)^* \right) \tag{S4}$$

where ϵ^{ijk} is the Levi-Civita tensor and corresponds to the vector product between the electric vector field and the induced dipole.

Supplementary Note 2

Optical Euler equations

The optical Euler equations describe the rotational dynamics of a rigid body subject to an optical torque. The Euler equations for a given rotational frame referenced torque is given by:

$$I_1 \frac{dp}{dt} = (I_2 - I_3)qr + \tau_1 \tag{S5}$$

$$I_2 \frac{dq}{dt} = (I_3 - I_1)rp + \tau_2 \tag{S6}$$

$$I_3 \frac{dr}{dt} = (I_1 - I_2)pq + \tau_3 \tag{S7}$$

where (I_1, I_2, I_3) are the three principal moments of inertia of the body in its own frame, (p, q, r) the three components of the angular velocity in the body frame and $\boldsymbol{\tau} = (\tau_1, \tau_2, \tau_3)$ the three torques. Using the angular velocity it is possible to define a rotation transformation matrix $\mathbf{R}$ linking the body fixed frame to the global frame. Its evolution is given by:

$$\frac{d\mathbf{R}}{dt} = \mathbf{R} \begin{pmatrix} 0 & -r & q \\ r & 0 & -p \\ -q & p & 0 \end{pmatrix}. \tag{S8}$$

The transformation matrix needs also to be used to represent the polarisability tensor and the electric field in the body frame. The optical dipole torque in the body frame is equal to

$$\boldsymbol{\tau} = \frac{1}{2}\mathbf{R}^{-1}\Re e\left((\mathbf{R}\boldsymbol{\alpha}\mathbf{R}^{-1}\mathbf{E}) \wedge \mathbf{E}^*\right). \tag{S9}$$

which in absence of rotation is equivalent to equation (S4).

Supplementary Note 3

Langevin equations

The Langevin equations describes the stochastic dynamics of the particle interacting with its environment:

$$m\frac{d\mathbf{v}}{dt} + \gamma\mathbf{v} \quad = \quad \mathbf{F} + \mathbf{f}^s \tag{S10}$$

where $\gamma = 6\pi\mu a$ corresponds to the drag coefficient (with μ the gas viscosity), $\mathbf{v}$ to the centre of mass velocity, m the mass of the particle, $\mathbf{F}$ and $\mathbf{F}^s$ the optical force (S3) and the stochastic force, respectively.

The stochastic force has a zero mean Gaussian distribution with the following properties:

$$< \mathbf{f}^s(t) > \quad = \quad 0 \tag{S11}$$

$$< f_i^s(t) f_j^s(t') > \quad = \quad 2k_B T\gamma\delta(t-t')\delta_{ij} \tag{S12}$$

where T is the temperature and k_B the Boltzmann constant.

Considering an optical harmonic potential defining the force $\mathbf{F} = -\kappa\mathbf{r}$ we can calculate the position power spectrum $S(\Omega/2\pi)$ by Fourier transforming Langevin equation:

$$S(\Omega/2\pi) = \frac{2\Gamma k_B T}{m}\frac{1}{(\Omega_0^2 - \Omega^2)^2 + \Gamma^2\Omega^2} \tag{S13}$$

where Ω is the angular frequency, $\Omega_0 = \sqrt{\kappa/m}$ the angular oscillation frequency and $\Gamma = \gamma/m$ the mass normalised drag coefficient. Integrating the position power spectrum leads to

$$\int_{-\infty}^{\infty} S(f)df = \langle x^2 \rangle = \frac{k_B T_{\text{eff}}}{m\Omega_\Gamma^2}, \tag{S14}$$

with $\Omega_\Gamma^2 = \Omega_0^2 - \Gamma^2/4$ and $2\pi f = \Omega$. Considering a drag coefficient much smaller then the natural oscillation frequency $\Gamma \ll \Omega_0$ we have:

$$\langle x^2 \rangle = \frac{k_B T_{\text{eff}}}{\kappa}, \tag{S15}$$

which shows that the msd $\langle x^2 \rangle$ is mass independent.

For mechanically anisotropic particles, the rotational Langevin equation [30] needs to be generalise by including an external torque[31] and contributions from the body frame

$$I_1\frac{dp}{dt} + \gamma_R p \quad = \quad (I_2 - I_3)qr + \tau_1 + \tau_1^s \tag{S16}$$

$$I_2\frac{dq}{dt} + \gamma_R q \quad = \quad (I_3 - I_1)rp + \tau_2 + \tau_2^s \tag{S17}$$

$$I_3\frac{dr}{dt} + \gamma_R r \quad = \quad (I_1 - I_2)pq + \tau_3 + \tau_3^s \tag{S18}$$

where $\gamma_R = 8\pi\mu a^3$ corresponds to the rotational drag coefficient and $\boldsymbol{\tau}^s$ the stochastic torque with the same properties as the translational stochastic force.

We consider briefly the influence of a periodic variation of the harmonic trapping potential force due to the rotation of the particle. In this case, the optical force is defined as $\mathbf{F} = -(\kappa + \epsilon\cos(\Omega_{\text{rot}}t))\mathbf{r}$ with ϵ the perturbation amplitude. The Fourier transform of the Langevin equation leads to a system of coupled equations:

$$\begin{aligned}\sqrt{2k_B T\Gamma m} \quad = \quad & \epsilon\, m\Omega_{\text{rot}}^2\hat{x}(\Omega - \Omega_{\text{rot}}) \\ & + (i\Gamma m\Omega + m(\Omega_0^2 - \Omega^2))\hat{x}(\Omega) \\ & + \epsilon\, m\Omega_{\text{rot}}^2\hat{x}(\Omega + \Omega_{\text{rot}})\end{aligned}$$

from which we can deduce the position power spectrum. These linear relationships can be solved in the first order with respect to the perturbation delivering a measure of the temperature at low frequency:

$$S(\Omega = 0) = \frac{2\Gamma k_B T}{m\Omega_0^4}\frac{(\Gamma^2 + (1 + 4\epsilon)\Omega_{\text{rot}}^2)}{(\Gamma^2 + \Omega_{\text{rot}}^2)} \tag{S19}$$

where we considered $\Omega_{\mathrm{rot}} \gg \Omega_0$. This highlights a first order correction term to the definition of the temperature. However, this term is independent of the rotation rate for $\Omega_{\mathrm{rot}} \gg \Gamma$. Indeed, Supplementary Figure S1 shows an example PSD, $S(f)$, signal for the case where $\Omega_{\mathrm{rot}} \gg \Omega_0$ and $\Omega_{\mathrm{rot}} \gg \Gamma$. Here, we observe the main peak corresponding to the trapping oscillation Ω_0 and two negligible peaks at the higher frequencies $\Omega_{\mathrm{rot}} + \Omega_0$ and $\Omega_{\mathrm{rot}} - \Omega_0$. These two peaks are related to the periodic perturbation. Due to their low amplitude, narrow width and high frequency position, these two peaks have a negligible contribution to the PSD signal at low frequency i.e. they do not affect the definition of the temperature using $S(\Omega = 0)$. A similar argument is valid when considering the integrated position power spectrum $\langle x^2 \rangle$ or stochastic high frequency perturbations.
